# Prevalence of Bifid Mandibular Condyle in a Selected Population in South of Iran

**Published:** 2014-12

**Authors:** Abdol Aziz Haghnegahdar, Pegah Bronoosh, Leila Khojastepour, Parissa Tahmassebi

**Affiliations:** a Dept. of Oral and Maxillofacial Radiology, School of Dentistry, Shiraz university of Medical Sciences, Shiraz, Iran.; b Undergraduate Student, School of Dentistry, International Branch of Shiraz University of Medical Sciences, Shiraz, Iran.

**Keywords:** Bifidity, Mandibular condyle, Panoramic radiography, Prevalence

## Abstract

**Statement of the Problem:** Bifid mandibular condyle (BMC) is a rare anomaly of uncertain origin which may play a role in some defects in temporomandibular joints. Since it may be misinterpreted as fractures or tumors in condylar area, proper diagnosis will help to prevent unnecessary treatments. A comprehensive knowledge about BMC may help to understand the developmental course of condyle and temporomandibular joints more clearly.

**Purpose:** The purpose of this study was to estimate the prevalence of bifidity of condyle in a selected population in Iran by employing dental panoramic view.

**Materials and Method:** Panoramic views of 1000 individuals (767 female, 233 male individuals) were assessed for bifidity. The patients were over 18 years old and had attended the radiology department of Shiraz dental school from September 2012 to March 2013.

**Results:** A total of 35(3.5%) case of bifidity was detected. Unilateral form was much more prevalent (32 unilateral cases versus 3 bilateral). The left-side bifidity was 3 times more prevalent than the right side. A large number of bifid condyles (63%) have shown symptoms of temporomandibular joint pain or click or both.

**Conclusion:** The prevalence of bifidity in our population was about 3.5%, which was significantly high compared to the other published reports. Symptoms (click and pain) were also much more detected in our study.

## Introduction

Bifid mandibular condyle (BMC, the presence of two heads over the neck of the condyle) was first described by Hrdlicka in 1941.[1] He reported 21 cases in a series of skull specimens. Sicher was the first researcher who reported a case of BMC in a living individual in 1948.[1] Morphologically, BMC might be restricted to a delicate notching on the condyle or extended as a complete lobulation of the condyles. Extensive division may result in two distinct heads while in less- completed cases; the heads are just separated by a shallow groove. The etiology of this entity is still controversial although two major theories have been postulated: traumatic origin and developmental anomaly. According to the first theory, a new condylar head may form in the space of traumatically broken and displaced condyle in response to the functional demands. On the other hand, remnants of embryonic fibrovascular septa in a developing condyle were considered as the major cause for developmental formation of two partially (or totally) separated heads. Endocrine disorders, deficiency of some nutrients, irradiation, infection, and genetic factors are also mentioned as the possible cause for BMC induction.[1-2] Severe remodeling of condyle which is associated with gross changes in the shape of condyle may also lead to BMC formation. Based on the position of the heads, BMCs are divided into two groups: those oriented mediolateraly and those located anteroposteriorly.[1-2] The orientation of the heads is defined by the origin of BMC; trauma-originated BMCs are mostly unilateral and anterior posteriorly located, while developmentally originated BMCs are most probably bilateral and mediolateraly in orientation. However, many of the cases are positioned obliquely in two different planes, which cannot be included in this simple classification.[1] Most of the cases are asymptomatic and are found accidentally, while some cases may present with pain and temporomandibular joint (TM joint) dysfunctions.

Many types of TM joint imaging are adopted for different diagnostic purposes including conventional radiographies, CT scan, MRI, ultrasonography, and cone beam CT scan. Conventional radiographies, including panoramic views, are the first line of diagnostic imaging of TM joint. They are low cost, easy-access techniques, and have relatively low dose exposure but suffer from superimpositions and distortions. The most common radiographic image is panoramic view which is prepared for most of the routine dental procedures. In this view, the image of TM joint partially loses its visibility because of the superimposition of the base of the skull. Since patient should slightly open the mouth during taking this view, the anatomic relationship of different parts of the joint will be mildly distorted.[3] In spite of these limitations, most of the BMC cases are detected in the panoramic views due to the widespread use of this imaging modality. CT scanning is a high-resolution cross-sectional imaging modality which is free of superimpositions and displays delicate structural changes. CT scanning can differentiate the tumor growth and the fracture lines but is associated with high doses of radiation exposure to the patient and high cost and less accessibility. A relatively long scanning time and complex equipment are also needed. Thus, CT scan is not considered as the primary imaging modality for TM joint. MRI serves as the gold standard for soft tissue imaging because of its highest contrast resolution. Although MRI can evaluate the disc position and morphology of TM joint, it is not the method of choice for evaluation of the osseous parts. Beside, long scanning time, high fee, claustrophobia, presence of metallic implants and pregnancy would limit the use of MRI in evaluation of TM joint in complicated cases or those needing confirmation of a suspected pathologic problem.[1-2] Sonography is a noninvasive real-time imaging technique. Images are constructed from the harmless reflected ultrasounds from the tissue. Regardless the advantages, the application of sonography for TM joint imaging is restricted at present time, although it has been used to determine the position of disc and form of the condyle in some researches.[4] Cone beam computerized tomography (CBCT) introduced a new generation of cross-sectional imaging modality which opened a new perspective in evaluation of anatomy and pathologic features in head and neck regions. The modality benefits from a cone-shaped beam and a receptor-area that captures all the desired data in a single rotation of the tube with a significantly decreased radiation dose to the patient. Panoramic, cross-sectional and three dimensional views can be reconstructed which helps in exact evaluation of the anatomic relationship between different parts of the subject. CBCT is increasingly used for TM joint imaging for inspection of the morphology of the condyle and glenoid fossa .It is also used in defining the anatomic relationship of different joint components or correlating the clinical status to the anatomic changes of the joint.[5] Today, CBCT is considered as a complementary view to evaluate the bony components and joint spaces in advanced TM joint examination which cannot replace the conventional views including panoramic views.

The diagnostic challenge is worthy since the radiographic detection of BMCs could be confused with fractures and tumors. BMC also seems to play a crucial role in some temporomandibular joint dysfunctions even though it has not yet been proved. This study is conducted to estimate the prevalence of BMC in a selected population in south of Iran, using panoramic radiography as a screening imaging tool. 

## Materials and Method

The panoramic views which were assessed in this cross-sectional study were taken from the patients who referred to the radiology department of Shiraz dental school for any reasons from September 2012 to March 2013. The views with unacceptable quality, the images taken from the patients younger than 18 years, and images exhibiting osseous diseases in condylar area were excluded from the study.

All the views were prepared using a Planmeca panoramic machine Prolin XC (Helsinky, Finland) by a Photo Stimulable Phosphor plate (PSP) (Konika Minolta; Tokyo, Japan), read by a laser reader (Reguis 110; Tokyo,Japan) displayed on a 17-inch, Flatron L1755SE, LG monitor (Tehran, Iran) with a resolution of 1280 * 1024.

All views were evaluated by a trained senior dental student on the monitor in a dark room using digital magnifying and searching for BMCs. 

Observation of two separate cortices for a condyle was considered as the sign of BMC. 

Those views, suspected to convey BMC, were evaluated under supervision of an oral and maxillofacial radiologist to confirm the bifidity of condyle. Demographic information, history of trauma or systemic disease, findings of the clinical exam such as pain, click, crepitus, limitation of mouth opening, and deviation were registered in a form coded for the patient. 

## Results

Panoramic views of 1017 patients were inspected for bifidity; 17 were excluded due to the unacceptable quality and superimpositions.

Among 767 female and 233 male patients, bifidity was detected in 35 individuals. Female and male patients showed 23 and 12 cases of bifidity respectively. Unilateral BMC comprised 32 cases while 3 cases were bilateral. 

24 BMC cases were detected on the left and 8 cases on the right side; the prevalence of BMC in the left side was more than the right side. 

The history of trauma in childhood was positive in about 14.3% (5 patients) of cases and 85.7% (30) of cases have mentioned negative history.

The click sign was detected in 12 cases of BMC and 10 cases suffered from pain and click on the movements. No cases have reported limitation of mouth opening, lateral excursions, or crepitus. The achieved data is summarized in Table 1.

**Table 1:** Distribution of BMCs

Patients		NO of patients with BMC		
1000		35		
M	F	Unilateral		Bilateral
233	767	32		3
	L	R	
	24	8	

## Discussion

The bifidity of the mandibular condyle is a rare finding. Most of the cases are found incidentally in panoramic views and the patients were unaware at the time of detection, although some had become symptomatic and interfered with function.

The real prevalence of BMC is still not clear and further epidemiologic studies are needed.[1, 3]

Most of the publications on BMC are case reports. In these reports, the BMC must be confirmed by a more reliable imaging technique such as CT scan or MRI. Although the images obtained from these modalities of imaging have high contrast and high resolution, they could not be employed in epidemiologic studies as the screening tools due to their high radiation doses, difficulty of preparing, time needed, and higher fees. On the other hand, panoramic view is a relatively low-dose exposure imaging modality with easy accessibility and low price which can yield a lot of information in a single view. Today most of the epidemiologic studies are conducted by employing panoramic views and we used these views which were taken for different dental diagnostic and treatment purposes, thus eliminating any extra radiation to the individuals. 

A study has reported an prevalence of 0.48% for BMC in 1882 examined dried skulls.[6] Living individuals would even show lower prevalences.[7-8]

In the English published literature, not more than 112 cases have been reported till 2011, although this number is rapidly increasing due to the use of new advanced imaging modalities. Many cases were reported in the last decade.[3]

It is noteworthy that two cases of trifidity of the condyle have been presented in 2003 and 2004.[3, 6]

BMC was more prevalent in left side.[6-8] Most of the cases were over 20 years old, although it can be seen in any ages.[3, 7, 9] Any sexual or racial differences are controversial for both unilateral and bilateral conditions. A minority of the involved individuals showed symptoms such as pain, swelling, and restriction in mouth opening, joint sounds, facial asymmetry, and ankylosis.

Most of the publications on BMC are case reports and epidemiologic studies are very insufficient.

Miloglu et al. reviewed 10200 panoramic projections and found an prevalence of 0.3% for BMC in a Turkish population. All cases were asymptomatic with no history of trauma or sexual difference. The unilateral BMCs were three times more prevalent.[7]

Menezes et al. found only 9 cases of BMC in 50080 panoramic views (0.018%), all were asymptomatic and no history of trauma was mentioned. The Unilateral cases (N=7) were more prevalent than the bilateral cases (N= 2) and more cases were detected in females (N=7) than the male patients (N=2).[8]

Sahman et al. reported an prevalence of 0.52% for BMC in Turkish population (98 cases in 18798 patients) with no difference in gender or affecting side; 72.4% were unilateral and 27.6% bilateral.[3]

Bong-Hae Cho et al. examined 3046 asymptomatic and 4378 patients with symptoms of TMJ dysfunction using CBCT. They detected 15 (0.49%) cases of BMCs in asymptomatic and 22(0.50%) cases of BMCs in symptomatic patients. There was neither any significant difference concerning the gender or the affected side nor any association with symptoms in this study.[10]

**Figure 1:**
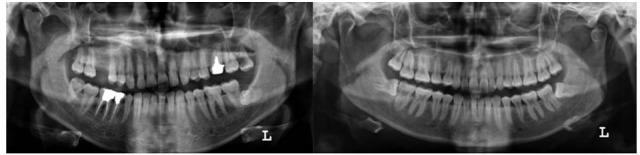
Conspicuous bifidity of condyle in panoramic views.

Comparing the results, the prevalence of BMC in our study (3.5%) was much higher than the other reports. It can be attributed to the racial differences or to the viewer’s judgment .We only considered the pointed and clear cases of BMC, and the suspicious cases were not included, those which could increase the prevalence rate even more if they were included as BMC (Figure 1). It is notable to say that the number of samples in our study was less than the other researches. It is worthy to mention that a clear and standard scale was not mentioned for BMC diagnosis in previous reports. The diagnosis of BMC was totally subjective with no quantification in all studies. Notably, an accentuated petrygoid fovea on the anterior surface of condyle which may extend to the superior aspects could be confused with BMC easily. No confirmed criteria were published for such differentiation. This might explain the different prevalence of BMC reported in different studies. As mentioned, the observation of two cortices on a condyle was considered as the first suspecting sign of BMC in this study.

Shahidi and Farnoodi[11] reported that 17% of TMD patients in Shiraz had BMC which was much more than 0.50% which was reported by Cho and Jung.[10] This assertion also shows that TMD is more associated with BMC in our tested population. The unilateral BMCs were more frequent in all studies with a ratio of about 3 to 1. Similarly in our study, unilateral cases were more than the bilateral cases but the ratio was not identical (10.6 to 1).

About two thirds of the cases of our study were symptomatic, suffering from click or pain or both. This ratio was compatible with the findings of the study conducted by Shahidi and Farnoodi but it is in contrast with the results obtained by Cho who reported the same prevalence in symptomatic and asymptomatic individuals.[10-11] This is also in contrast with the results of Miloglu in Turkey and Menezes in Brazil whose cases were all asymptomatic.[7-8] It may be attributed to the different ethnicity of the studied population or different diagnostic criteria employed in their study. Moreover, the high rate of car accident in the area of study may play a role in higher prevalence of trauma-originated BMC in our study.

The symptoms may be present in all types of BMC and many factors may influence the presence or absence of symptoms. It should be proposed that the etiology of the BMC maybe relatively responsible for the symptoms. It seems logic that traumatic BMCs show a higher rate of symptoms because trauma is usually associated with pain, inflammation, and function disturbance during a short period of time. Meanwhile, developmental BMCs may more probably be asymptomatic, since the involved individual has the chance to adapt the situation through the relatively long period of development and growth. This hypothesis must be evaluated by future researches.

The history of trauma was present in 14.3% of cases in our study which is lower than what was mentioned in literature. This is probably because of the limited number of samples in the present study. Finally, we believe that BMC is significantly more prevalent in our population according to the results obtained by the present study and Shahidi et al.’s. Further investigation is needed to assess the relationship between BMC and TMDs if exists. 

## Conclusion

The prevalence of BMC in studied population was (3.5%) which was significantly higher than the previous reports. Most of the cases were unilateral and left sided. Pain and click were the prevalent symptoms in BMC cases in the tested population. 
